# Rhythmic expression of circadian clock genes in the preovulatory ovarian follicles of the laying hen

**DOI:** 10.1371/journal.pone.0179019

**Published:** 2017-06-12

**Authors:** Zhichao Zhang, Shuang Lai, Yagang Wang, Liang Li, Huadong Yin, Yan Wang, Xiaoling Zhao, Diyan Li, Mingyao Yang, Qing Zhu

**Affiliations:** 1 Institute of Animal Genetics and Breeding, Sichuan Agricultural University, Sichuan, Chengdu, China; 2 College of Animal Science and Technology, Sichuan Agricultural University, Sichuan, Chengdu, China; Kent State University, UNITED STATES

## Abstract

The circadian clock is reported to play a role in the ovaries in a variety of vertebrate species, including the domestic hen. However, the ovary is an organ that changes daily, and the laying hen maintains a strict follicular hierarchy. The aim of this study was to examine the spatial-temporal expression of several known canonical clock genes in the granulosa and theca layers of six hierarchy follicles. We demonstrated that the granulosa cells (GCs) of the F1-F3 follicles harbored intrinsic oscillatory mechanisms *in vivo*. In addition, cultured granulosa cells (GCs) from F1 follicles exposed to luteinizing hormone (LH) synchronization displayed *Per2* mRNA oscillations, whereas, the less mature GCs (F5 plus F6) displayed no circadian change in *Per2* mRNA levels. Cultures containing follicle-stimulating hormone (FSH) combined with LH expressed levels of *Per2* mRNA that were 2.5-fold higher than those in cultures with LH or FSH alone. These results show that there is spatial specificity in the localization of clock cells in hen preovulatory follicles. In addition, our results support the hypothesis that gonadotropins provide a cue for the development of the functional cellular clock in immature GCs.

## Introduction

The circadian clock is a cell-autonomous system that coordinates physiology and metabolism to the correct time of the day [1, 2]. The endogenous timekeeper is based on intracellular transcriptional-translational feedback loops in which a few canonical clock gene products and genetic elements drive the rhythmic expression of downstream genes, thereby contributing to rhythmic physiology [3–5]. The circadian system is important for successful reproduction in vertebrates because it influences the follicle maturation and ovulation [6].

The avian central circadian organization consists of three separate oscillators located in the pineal gland, the retinae and a hypothalamic region, that is possibly equivalent to the mammalian SCN [7–9]. Prior studies have supported the intimate relationship between the circadian timing system and the hypothalamic-pituitary-gonadal (HPG) axis. Briefly, a signal from the central clock is crucial for the initiation of the luteinizing hormone (LH) surge and subsequently, for ovulation. Recently, the ovarian circadian clock has been well documented in many mammalian and non-mammalian species, and its function has been investigated both *in vivo* and *in vitro*. In most known cases, the function of the ovarian clock is related to the timing of gene expression in mature granulosa cells (GCs), including genes related to steroidogenesis, gonadotropin responsiveness and ovulation [10].

Clock gene rhythms are possibly under the control of gonadotropins (follicle-stimulating hormone (FSH) and LH), acting to synchronize of follicular cell activities [11–14]. On the other hand, several candidate genes associated with the response of GCs to gonadotropins are known as clock-controlled genes (CCGs), such as the LH receptor (*Lhcgr*), steroidogenic enzymes (*StAR*), prostaglandin synthase (*ptgs2*) and gap junctions [15–17]. Obviously, there is a correlation and interaction between the presence of a functional clockwork and gonadotropin-induced follicular development. However, it is difficult to determine whether the activation of the ovarian clock is a cause or a result of follicle development and maturation.

The ovary is an ever-changing, multi-compartmental organ. In chickens, a strict follicular hierarchy is maintained, with 2 to approximately 6 hierarchical follicles (F1-F6) and a single follicle ovulated per day [12, 18–20]. In quails, Nakao found that the level of *StAR* mRNA exhibited a diurnal rhythm coincident with *Per2* expression only in the largest follicle [21]. Therefore, gonadotropins and their associated cell signaling mechanisms combined with the ovarian clock might orchestrate a functional hierarchy of preovulatory follicles. To further understand the role of the ovarian clock during the maturation of follicles, a thorough analysis of clock gene expression in preovulatory follicles is needed to determine whether the rhythm is truly circadian and to reveal the detailed expression patterns in the specific components of different follicles [22].

Birds represent an excellent model to study the role of biological clocks in female reproduction for two reasons: ① The daily rhythms of ovulation-oviposition in adult female birds have been well described and are easy to monitor [23, 24]. ② The morphological characteristics and functional anatomical components of preovulatory follicles are well known, and the stage of follicular development is defined by the follicle size. Accordingly, the present study explored differences in spatial-temporal relationships among clock genes in GCs and theca cells (TCs). Moreover, on the basis of the present results, we examined the effects of gonadotropins on *Per2* mRNA expression, providing preliminary support for the hypothesis that FSH provides a cue for the development of the functional cellular clock in immature GCs, whereas LH synchronizes the cellular clock of mature GCs.

## Materials and methods

### Ethics statement

All animal experiments were approved by The Committee on Experimental Animal Management of Sichuan Agriculture University, and carried out in strict accordance with the Regulations for the Administration of Affairs Concerning Experimental Animals of the State Council of the People’s Republic of China. The chickens involved in this study were sacrificed with as little pain as possible to reduce their suffering.

### Animals and Zeitgeber Time (ZT)

Twenty-week-old Roman laying hens were housed individually in an experimental farm for poultry breeding at the Sichuan Agricultural University (Ya’an, China) and provided feed and water *ad libitum*. The hens were exposed to cycles of 16 h of light (L) and 8 h of darkness (D), with the light period beginning at 06:00 h local time. ZT0 was defined as the time at which the lights came on. As described previously [25], adult birds (20–30 weeks of age) with regular clutches of at least 30 eggs were used for these experiments. Individuals or groups of chickens that were collected at specific times of the circadian day were sacrificed for RNA extraction. This procedure made it impossible to analyze the molecular rhythms within individuals over successive time points. Thus, we monitored individual daily laying patterns by assessing the oviposition time of each bird at 30-min intervals from ZT0 to ZT16. After 4 weeks of ovulatory cycle observations, 24 birds that had consistent laying sequences and oviposition times (between ZT2 and ZT2.5) were selected from among the 500 individuals for further experiments.

### Tissue collection and GC culture

Selected hens were anesthetized with pentobarbital and sacrificed by decapitation at 2, 6, 10, 14, 18, and 22 hours after oviposition (n = 3 per time point). The experimental procedures were approved by the Animal Care Committee at our institute. After sacrifice, the ovary from each hen was immediately removed and placed into an ice-cold 0.9% NaCl solution. TCs and GCs were collected from large preovulatory follicles (F1-F6) according to previously described methods [26, 27]. Tissues were immediately snap frozen in liquid nitrogen and stored at -80°C until RNA extraction.

For culture, hens were sacrificed 2 h after oviposition. Follicles were arranged according to size. GCs from F1 follicles and F5 plus F6 follicles (F5/F6) were studied separately. The isolated sheets were washed in pre-warmed Hanks’ balanced salt solution to remove the adherent yolk, cut into pieces and dissociated in Type IV collagenase (1.5 mg/mL, Sigma, St Louis, MO, USA) containing M199 medium (Gibco BRL Co.Ltd., USA). The viable dispersed cells were enumerated using the trypan blue exclusion method and suspended in M199 medium containing 5% fetal bovine serum (FBS) and a 1% penicillin-streptomycin mixture. Cells were cultured at 37°C in a humidified atmosphere of 95% air and 5% CO_2_. Approximately 0.5 × 10^6^ GCs were seeded onto 12-well plates (Corning, NY, USA). After 24 h of incubation with a medium change at 6 h, the non-attached cells were removed by aspiration, and the adherent cells were washed three times with serum-free medium and used in all further incubations. Ovine LH (100 ng/mL), and recombinant human FSH (100 ng/mL) were dissolved in culture media.

### RNA extraction, reverse transcription and Real-Time PCR analysis

Total RNA was extracted from the tissues using TRIzol reagent (Invitrogen, Carlsbad, CA, USA) according to the manufacturer’s instructions. For reverse transcription, cDNA was synthesized from 1 μg of total RNA with the PrimeScript^®^ RT Master Mix (Perfect Real Time) (TaKaRa, Dalian, China) following the manufacturer’s protocol. All qPCR assays were performed on a Bio-Rad CFX96 Touch system (Bio-Rad, Hercules, CA, USA). Each reaction volume was 15 μl, containing 1 μl of cDNA, 0.5 μl of the reverse and forward primers (10 μM) for each gene, 5.5 μl of double-distilled H_2_O, and 7.5 μl of the SsoFast Eva Green Supermix (Bio-Rad, Hercules, CA, USA). Triplicate no-template controls containing DEPC-treated water were included in each run. All of the primers employed in this study were published previously [25]. Primer sequence details are provided in Supplementary S1 Table. Relative expression was determined using the 2^-ΔΔCT^ method. The quantity in each sample was measured relative to β-actin.

### Statistical analysis

Data are presented as the mean ± SEM of at least three separate experiments. To detect the rhythmically expressed genes, we used 2 different algorithms. Cosinor analysis adjusts the data to a cosinusoidal function and provides an objective test of whether the amplitude of the cosinor curve differs from zero [28]. For this purpose, we fitted periodic sinusoidal functions to the relative values for the expression of each gene across six time points (over a 24-h period) using the formula ⨍(*t*) = *M* + *A cos*(2*πt*/24 + *σ*), where ⨍(*t*) is the level of gene expression across time, the mesor *M* is the middle value of the fitted cosine representing a rhythm-adjusted mean, *A* is the amplitude of the oscillation in expression, *t* is the time in hours, and *σ* is the acrophase (the peak time of the fitted cosine function). In addition to the performing the cosinor analysis, we analyzed the data using CircWave software (R.A.Hut, Groningen, NL) [29]. CircWave uses a linear harmonic regression fit that describes the data by adding harmonics to the principal wave function. To determine the number of harmonics to add, F-testing was used for the primary fit and for each added harmonic, with a significance level of 0.001 adopted to reduce the chance of false positives. One-way ANOVA and Student’s *t*-test were also performed, using SigmaPlot software (Ver. 12.0; Systat Software, San Jose CA) and GraphPad Prism. Differences were considered significant at P < 0.05.

## Results

### Circadian clock gene expression in preovulatory follicles *in vivo*

The mRNA of seven clock genes (*Bmal1*, *Bmal2*, *Clock*, *Per2*, *Per3*, *Cry1* and *Cry2*) was expressed at all time points in the preovulatory follicles of laying hens maintained under a 16:8 L: D schedule. The analysis of mRNA expression revealed that most of the genes showed diurnal changes, but not all of the clock gene transcripts were expressed rhythmically. The expression and rhythm characteristics of all clock genes in GCs and TCs are summarized in Table 1. As shown in the table, clock genes that showed pronounced circadian rhythms in expression were more common in GCs than in TCs, and the rhythm-adjusted 24-h means of the expression levels of most clock genes were higher in GCs than in TCs.

**Table 1:** Circadian rhythm characteristics of clock genes in GCs and the TCs.

	Mesor	Amplitude	Acrophase(hh:mm)	*P*-value	Mesor	Amplitude	Acrophase(hh:mm)	*P*-value
F1	Granulosa				Theca			
*Bmal1*	5.36	2.67	13:35	<0.001	0.33	0.08	15:28	<0.001
*Bmal2*	0.15	0.02	05:43	>0.05	0.70	0.15	04:04	>0.05
*Clock*	0.68	0.30	15:27	<0.001	1.01	0.30	20:44	<0.05
*Per2*	5.67	7.00	06:41	<0.001	0.59	0.21	07:53	<0.05
*Per3*	0.38	0.12	06:46	<0.05	0.05	0.05	23:02	<0.05
*Cry1*	2.39	2.04	18:15	<0.01	0.24	0.08	02:42	>0.05
*Cry2*	3.49	2.02	06:54	<0.001	0.89	0.23	22:24	>0.05
F2	Granulosa				Theca			
*Bmal1*	5.39	2.45	16:45	<0.001	0.42	0.07	17:36	>0.05
*Bmal2*	0.27	0.36	23:53	<0.001	0.85	0.09	06:16	>0.05
*Clock*	1.53	2.01	23:57	<0.001	1.01	0.09	01:18	<0.05
*Per2*	3.23	2.06	08:19	<0.001	0.63	0.09	08:36	>0.05
*Per3*	0.51	0.55	23:40	>0.05	0.03	0.01	07:14	>0.05
*Cry1*	1.68	1.16	20:13	>0.05	0.23	0.03	20:36	>0.05
*Cry2*	3.27	1.69	02:30	>0.05	1.13	0.56	15:39	<0.001
F3	Granulosa				Theca			
*Bmal1*	4.85	1.41	13:08	<0.05	0.32	0.07	12:10	>0.05
*Bmal2*	0.18	0.15	22:06	>0.05	0.77	0.29	03:49	<0.05
*Clock*	0.40	0.17	18:52	<0.001	0.76	0.25	03:26	<0.01
*Per2*	1.38	1.10	08:14	<0.001	0.44	0.19	06:56	<0.01
*Per3*	0.13	0.06	10:25	>0.05	0.02	0.01	08:15	<0.01
*Cry1*	0.70	0.18	06:24	>0.05	0.17	0.03	01:48	>0.05
*Cry2*	2.38	1.09	06:40	<0.001	0.58	0.09	03:40	>0.05
F4	Granulosa				Theca			
*Bmal1*	5.26	1.76	16:06	>0.05	0.41	0.05	09:15	<0.05
*Bmal2*	0.94	1.64	18:41	<0.05	1.05	0.26	03:46	>0.05
*Clock*	1.14	1.45	06:05	<0.05	0.81	0.11	06:53	<0.05
*Per2*	1.75	0.81	11:33	<0.01	0.61	0.40	09:27	<0.01
*Per3*	0.14	0.05	13:05	>0.05	0.05	0.05	13:27	<0.01
*Cry1*	1.01	0.73	15:36	<0.05	0.22	0.07	17:45	<0.05
*Cry2*	3.91	2.24	18:42	<0.01	0.71	0.04	17:13	>0.05
F5	Granulosa				Theca			
*Bmal1*	6.06	0.55	23:10	>0.05	0.46	0.06	09:26	>0.05
*Bmal2*	0.42	0.11	19:24	<0.05	1.01	0.16	05:02	>0.05
*Clock*	0.67	0.06	06:05	>0.05	0.72	0.06	19:28	>0.05
*Per2*	2.74	0.76	06:35	<0.05	0.35	0.19	11:07	<0.01
*Per3*	0.18	0.02	16:39	>0.05	0.02	0.00	13:16	<0.05
*Cry1*	1.37	0.53	16:42	>0.05	0.18	0.02	17:55	>0.05
*Cry2*	5.14	0.59	15:16	>0.05	0.63	0.01	00:30	>0.05
F6	Granulosa				Theca			
*Bmal1*	2.44	1.10	19:41	<0.01	0.83	0.20	03:57	<0.01
*Bmal2*	0.62	0.23	14:08	<0.05	1.33	0.27	03:31	>0.05
*Clock*	0.72	0.50	18:32	<0.001	1.37	0.14	05:40	>0.05
*Per2*	6.12	1.90	16:53	<0.01	0.84	0.40	06:44	<0.001
*Per3*	0.42	0.12	16:37	>0.05	0.06	0.02	10:00	<0.05
*Cry1*	0.37	0.09	17:21	>0.05	0.37	0.07	13:24	<0.05
*Cry2*	6.84	3.05	16:42	<0.001	1.17	0.09	02:03	>0.05

The acrophases and amplitudes were calculated according to the non-linear regression fit of a cosine function. CircWave was used to detect a significant rhythm, and *P* < 0.001 was considered statistically significant.

**Fig 1:**
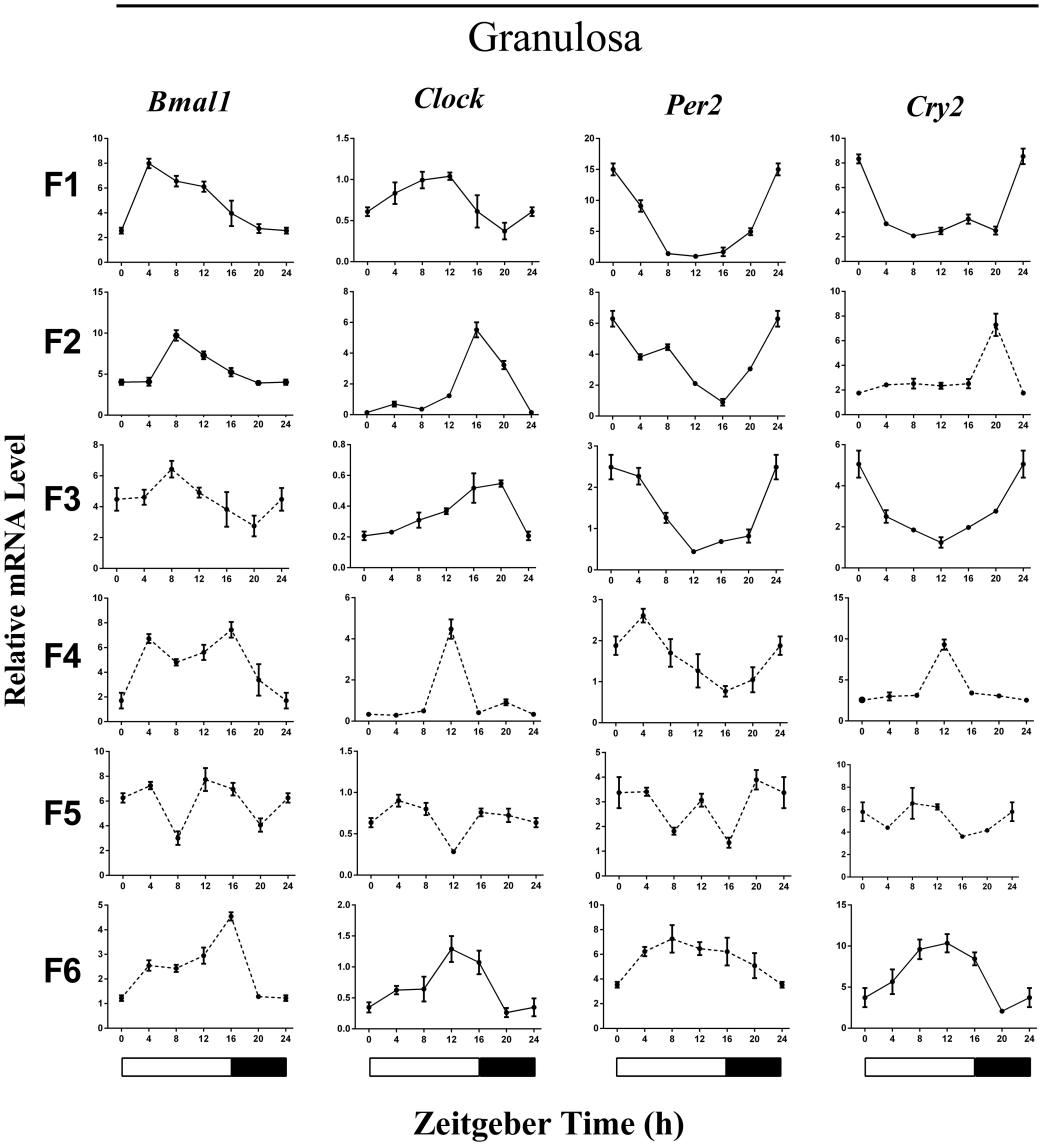
Daily expression patterns of selected clock genes in GCs of various preovulatory follicles under 16L:8D. Each data point represents the mRNA amount of the corresponding clock gene normalized to *β-actin* expressed as the mean ± SEM (n = 3). The white areas of the bars in the bottom of the figure indicate the light period, and the black areas indicate the dark period. ZT24 values are a duplicate of ZT0 shown for clarity. Genes were identified as rhythmically expressed in unison by CircWave, *P* <0.001 was considered to indicate significant rhythmic expression (solid lines). The dotted lines indicate no rhythmic expression.

The normalized daily *Bmal1*, *Clock*, *Per2* and *Cry2* mRNA expression profiles are conventionally used as circadian phase markers and are presented in Fig 1. The phase markers exhibited rhythmic expression with the expected phase alignments in the GCs of the F1-F3 follicles. For example, the *Bmal1* (positive element) and *Per2* (negative element) mRNA levels cycled in antiphase (Fig 1). In contrast, the statistical analysis revealed that no or few clock genes showed significant circadian variation in the TCs, and none of the expected phase alignments were observed in any of the examined follicle TCs (S1 Fig).

### Analysis of clock gene expression in cultured GCs

**Fig 2:**
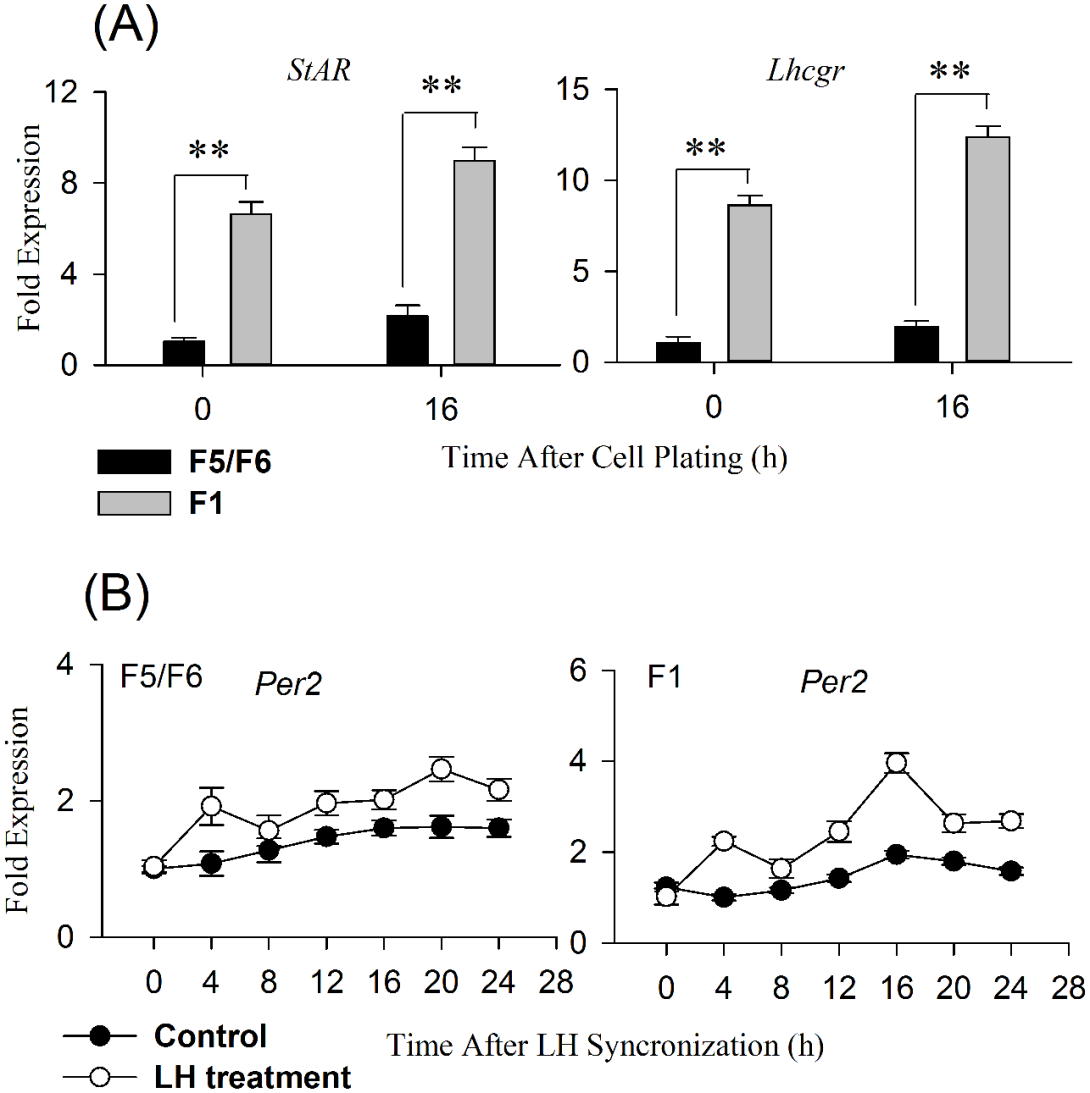
A: Differences in the expression of *Lhcgr* and *StAR* between F1 and F5/F6 GCs. B: *Per2* transcript profile in F1 and F5/F6 GCs synchronized by LH. GCs were synchronized for 2 h with 100 ng/ml ovine LH and then washed two times with serum-free medium.

Preovulatory follicle GCs were divided into two groups (F1 and F5/F6). Expression levels of the *Lhcgr* and *StAR* genes, as markers of GCs maturation, were significantly higher in F1 GCs than in F5/F6 GCs (Fig 2A). When the cells were cultured in a serum-free medium for 2 days, no or very weak oscillating mRNA expression was detected in all of the cultured cells. After LH treatment, circadian oscillation of *Per2* expression was clearly observed in F1 GCs (CircWave, *P* <0.001), whereas only time-dependent variations in mRNA levels were observed in cultured F5/F6 GCs (Fig 2B).

### Alterations of *Lhcgr* and *Per2* expression upon LH and FSH stimulation

**Fig 3:**
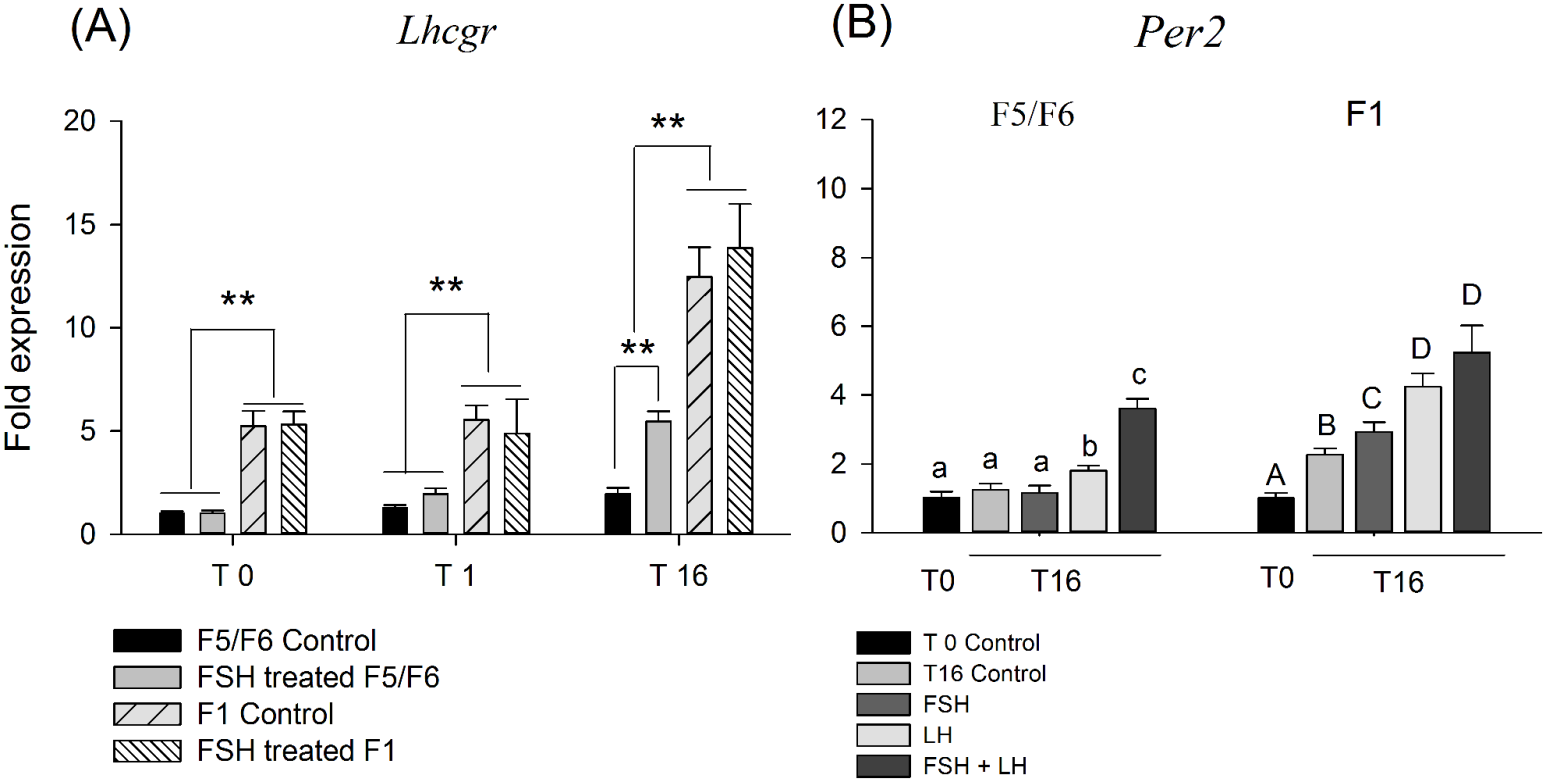
A: FSH-mediated induction of *Lhcgr* mRNA expression in GCs from F5/F6 and F1 follicles. B: Gonadotropin-mediated induction of *Per2* mRNA expression in GCs from F5/F6 and F1 follicles after a 16-h culture. Cells were plated in the absence (Control) or presence of recombinant human FSH (100 ng/ml) for 1 h (T1) or 16 h (T16). *: *P* < 0.05, **: *P* < 0.01. The data represent the mean fold difference (± SEM) *vs*. T0 (*Per2* mRNA) cultured cells from three replicate experiments. a, b, c; A, B, C, D: *P* < 0.05.

We investigated the expression of *Lhcgr* and *Per2* in cultured F1 and F5/F6 GCs. In the F5/F6 group, the levels of *Lhcgr* mRNA were increased after treatment with FSH for 1 h (1.6 ± 0.13-fold compared with the T1 control) and remained elevated and further increased after 16 h of culture (5.5 ± 0.7-fold T16 control), whereas a significant increase in response to FSH treatment was not observed in the F1 follicle GCs. However, the levels of *Lhcgr* mRNA were markedly lower in F5/F6 follicles GCs than in F1 follicles regardless of FSH-treat (Fig 3A). As shown in Fig 3B, time-dependent variation in the mRNA levels of *Per2* was observed in the F1 GCs but not F5/F6 GCs. Both FSH and LH stimulation significantly increased the levels of *Per2* mRNA in F1 GCs compared with the no-treatment controls (*P* < 0.05); however, FSH treatment did not affect the levels of *Per2* mRNA expression in F5/F6 GCs (*P* > 0.05). Treatment with FSH plus LH induced a significant increase in *Per2* expression in the cultured cells from both the F5/F6 and F1 groups compared with the controls (3.6 ± 0.28-fold and 5.2 ± 0.78-folds, respectively).

## Discussion

Ovarian preovulatory follicles also known as hierarchical follicles (F1-F6) are selected from the small yellow follicles (SYF) and are destined to ovulate; the F1 follicle will be the next to ovulate. Oviposition is followed 15–45 min later by ovulation, after which each follicle moves up one position in the hierarchy. The GCs of different follicles are at different levels of differentiation, and steroidogenic ability differs among the six preovulatory follicles.

In the present study, we showed the cyclical gene expression patterns of several canonical clock genes in ovarian preovulatory follicles during the ovulation-oviposition cycle (~24 h) of commercial hens under farming light-dark conditions. Typically, the expression of a positive element (*Bmals* and *Clock*) in an antiphase relationship to the negative elements (*Pers* and *Crys*) in any given tissue is considered evidence of a functional clock [30]. In a previous study, Nakao *et al*. [21] found a robust change over 24 h in *Per2* and *Per3* gene expression only in mature F1 follicles based on the Fisher’s least significant difference (LSD) post hoc test. In our study, the data were analyzed using CircWave 1.4 software [29] to test the daily rhythmicity of gene expression. We found that several of the clock genes exhibited rhythmic expression (*P* < 0.001), with the expected phase alignments in the GCs of the F1, F2 and F3 follicles (Fig 1). Although several clock genes also appeared to change over 24 h in other tissues (e.g., in the GCs of F6 follicles), no significant rhythmic pattern or anticipated antiphasic relationship was observed (Fig 1). Our results provide a thorough and precise analysis of clock gene expression in preovulatory follicles and reveal tissue-specific variations in phase and amplitude. These findings are in agreement with those of previous studies suggesting that the specificity of clock gene oscillations may be fine-tuned to functional compartmentalization of the preovulatory follicles.

On the basis of our present understanding of the spatial specificity in the localization of clock cells in the hierarchal follicles, we cultured GCs from F1 and F5/F6 follicles synchronized with LH. LH is a potent synchronization factor *in vitro* as described in previous studies [16, 31–33]. Our results showed that *Per2* oscillations were generated by LH synchronization only in GCs from F1 follicles; the clock genes of less mature GCs prepared from F5/F6 follicles displayed no circadian pattern. The present findings are consistent with previous reports of much more active cellular clock in matured GCs than in less mature GCs [16, 21, 32]. LH plays various roles through combination with its receptor (*Lhcgr*). *Lhcgr* mRNA is first present in the granulosa layer of the smallest preovulatory follicles and increases with follicular maturation [34, 35]. Thus, it is possible that the elevation of *Per2* oscillations in F1 GCs may be due to the increased transcription of *Lhcgr*. In this study, the *Lhcgr* mRNA level was statistically significantly higher in cultured GCs from F1 follicles than in those from F5/F6 follicles (Fig 2A). LH-stimulated *Per2* gene expression may be dependent on high levels of *Lhcgr* expression.

FSH treatment upregulated the expression of *Lhcgr* mRNA in GCs from F5/F6 follicles (Fig 3A). Of the core clock genes, *Pers* (*Per1*-*Per3*) are the most responsive to various inducers *in vivo* or *in vitro*. However, FSH treatment had no effect on the expression of *Per2* in F5/F6 GCs. In contrast, a slight but significant increase in *Per2* mRNA levels was observed after LH treatment. Interestingly, FSH plus LH treatment was most effective in increasing the expression of *Per2* mRNA (Fig 3B). We failed to observe oscillatory expression of *Per2* in FSH-treated GCs by LH synchronization.

Based on work performed by the present authors and others, one hypothesis is that FSH can induce the sustained increase of *Lhcgr*, which is accompanied with follicular development, and alter the responsiveness of follicles to LH as a result. The high-responsiveness follicles may be synchronized by the pulsatile LH surge, leading to a gradual activation of the functional clock in follicles. Furthermore, the follicular clock system maximizes the expression of LH receptors preceding the arrival of the LH surge. Our data, consistent with several previous reports, showed that there was spatial specificity in the localization of clock cells in the preovulatory follicles. Our results support the hypothesis that FSH provides a cue for the development of the functional cellular clock in immature GCs, and that LH synchronizes the cellular clock of mature GCs. This work contributes to our understanding of the physiological significance of rhythmic clock gene expression in ovarian follicles.

## Supporting information

S1 Fig Daily expression patterns of selected clock genes in TCs of various preovulatory follicles under 16L:8D. Each data point represents the mRNA amount of the corresponding clock gene normalized to *β-actin* expressed as the mean ± SEM (n = 3). The white areas of the bars in the bottom of the figure indicate the light period, and the black areas indicate the dark period. ZT24 values are a duplicate of ZT0 shown for clarity. Genes were identified as rhythmically expressed in unison by CircWave, *P* <0.001 was considered to indicate significant rhythmic expression (solid lines). The dotted lines indicate no rhythmic expression.
(TIF)

S1 Table Real-time PCR primer sequences for the clock genes. (DOCX)
