# Supplementary figures and images for: Rhythmic expression of circadian clock genes in the preovulatory ovarian follicles of the laying hen
Source: PLoS One. 2017 Jun 12;12(6):e0179019. doi: 10.1371/journal.pone.0179019 (PMC5467841; doi:10.1371/journal.pone.0179019)

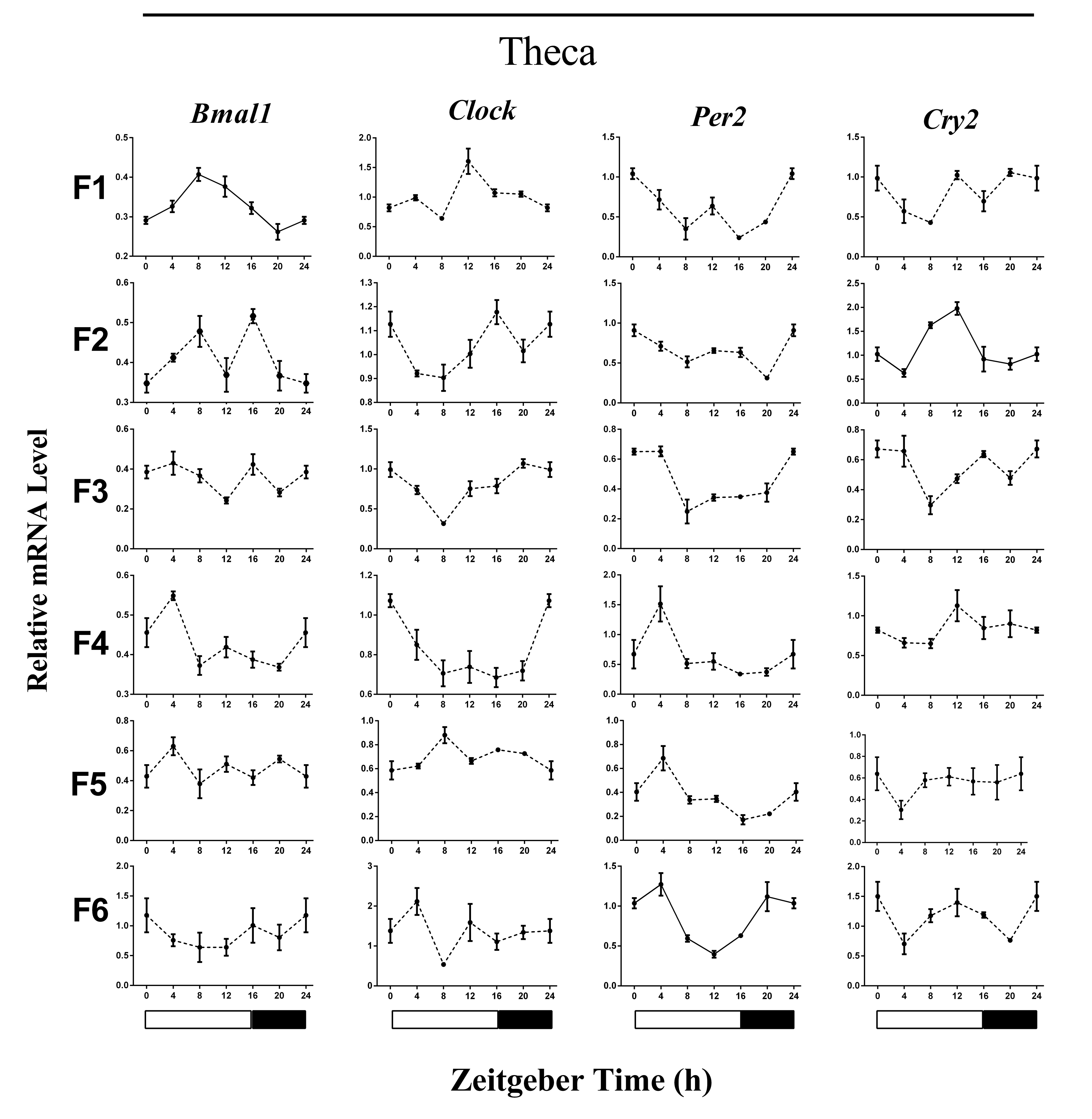

Supplement: S1 Fig — Each data point represents the mRNA amount of the corresponding clock gene normalized to β-actin expressed as the mean ± SEM (n = 3). The white areas of the bars in the bottom of the figure indicate the light period, and the black areas indicate the dark period. ZT24 values are a duplicate of ZT0 shown for clarity. Genes were identified as rhythmically expressed in unison by CircWave, P <0.001 was considered to indicate significant rhythmic expression (solid lines). The dotted lines indicate no rhythmic expression. (TIF) [file pone.0179019.s001.tif]
