# Supplementary material for: Rhythmic expression of circadian clock genes in the preovulatory ovarian follicles of the laying hen
Source: PLoS One. 2017 Jun 12;12(6):e0179019. doi: 10.1371/journal.pone.0179019 (PMC5467841; doi:10.1371/journal.pone.0179019)
Supplement: S1 Table — (DOCX) [file pone.0179019.s002.docx]

| **Gene** | **GenBank** | **Sequence 5’ - 3’** | **bp** |
| --- | --- | --- | --- |
| *cBmal1* | [NM_001001463](http://www.ncbi.nlm.nih.gov/nuccore/NM_001001463.2) | F: CTGTGACAGAGGGAAGATACTGT | 119 |
|  |  | R: TGGCAATGTCTTTCGGATGAAGG |  |
| *cBmal2* | NM_204133 | F: ATGCACAACAACCGAACATC | 316 |
|  |  | R: CAATGTTGTCCCATTTGAAGC |  |
| *cClock* | [NM_001289834](http://www.ncbi.nlm.nih.gov/nuccore/NM_001289834.1) | F: TGAGCTCCATTGCAGACAGAA | 178 |
|  |  | R: TCCATCTTCCGAGCATTACCT |  |
| *cPer2* | [NM_204262](http://www.ncbi.nlm.nih.gov/nuccore/NM_204262.1) | F: CCAACTTTGTTGTGTGCTCCTTGC | 115 |
|  |  | R: CTGGAACAAACAGGTTGGTGTGTG |  |
| *cPer3* | NM_001289779 | F: CACAGGTTCAGCAGCATCAG | 219 |
|  |  | R: ATCATCACCCAGGCTGACTC |  |
| *cCry1* | NM_204245 | F: CCGGGAAACGCCCAAA | 66 |
|  |  | R: TGCTCTGCCGCTGGACTT |  |
| *cCry2* | [NM_204244](http://www.ncbi.nlm.nih.gov/nuccore/NM_204244.1) | F: ACAAGCCACCCCTCACCTAC | 150 |
|  |  | R: GATGGGACCCCATACACATC |  |
| *cβ-actin* | NM_205518 | F: TGCGTGACATCAAGGAGAAG | 111 |
|  |  | R: GACCATCAGGGAGTTCATAGC |  |
